# Phase angle associates with severity and mortality in acute-on-chronic liver failure

**DOI:** 10.3389/fmed.2025.1541795

**Published:** 2025-07-21

**Authors:** Shiran Cai, Liqun Lin, Yanyan Cai, Chenhao Wang, Yufen Lin, Jingping Zhou, Fei Zhou, Meiya Chen

**Affiliations:** ^1^Department of Gastroenterology, The National Key Clinical Specialty, Zhongshan Hospital of Xiamen University, School of Medicine, Xiamen University, Xiamen, Fujian, China; ^2^The Graduate School of Fujian Medical University, Fuzhou, China; ^3^Department of Gastroenterology, The Second Afliated Hospital of Fujian Medical Uiversity, Quanzhou, Fujian, China; ^4^Department of Clinical Nutrition, Zhongshan Hospital of Xiamen University, School of Medicine, Xiamen University, Xiamen, Fujian, China; ^5^Department of Oncology, Cancer Hospital, Fudan University (Xiamen Branch), Xiamen, Fujian, China; ^6^Institute for Microbial Ecology, School of Medicine, Xiamen University, Xiamen, Fujian, China; ^7^Department of Digestive Disease, School of Medicine, Xiamen University, Xiamen, Fujian, China; ^8^Xiamen Key Laboratory of Intestinal Microbiome and Human Health, Zhongshan Hospital of Xiamen University, School of Medicine, Xiamen University, Xiamen, Fujian, China

**Keywords:** acute-on-chronic liver failure, phase angle, prognosis, inflammation, bioelectrical impedance analysis

## Abstract

**Background:**

Acute-on-chronic liver failure is characterized by acute hepatic decompensation and high short-term mortality, thereby necessitating prompt prognostic assessment. Although phase angle (PhA) has been established as a biomarker in chronic diseases, its prognostic significance in ACLF remains unclear.

**Methods:**

In this study, we evaluated PhA in 78 ACLF patients and compared the results with those of two control groups: 45 patients with chronic hepatitis B infection but normal liver function, and 51 patients with abnormal liver function who did not meet the ACLF criteria. Upon hospital admission, comprehensive laboratory parameters were obtained, and PhA measurements were conducted to explore the associations among PhA, organ dysfunction indices, and established prognostic scoring systems for predicting 90-days outcomes in ACLF patients.

**Results:**

Our analysis demonstrated that ACLF patients exhibited significantly lower PhA values compared with both control groups. Notably, non-survivors within 90 days had substantially lower PhA levels than survivors. Additionally, patients with complications, including hepatic encephalopathy, ascites, gastrointestinal bleeding, and infection, showed markedly lower PhA values than those without such complications. Moreover, the combination of PhA with the Chronic Liver Failure - Sequential Organ Failure Assessment (CLIF-SOFA) score enhanced the predictive accuracy of 90-days mortality in ACLF patients.

**Conclusion:**

Phase angle serves as a valuable biomarker for evaluating ACLF severity and predicting short-term mortality, potentially offering a novel approach to risk stratification in ACLF management.

## Introduction

Chronic liver diseases may induce systemic inflammation through multiple pathways, including pathogen invasion and tissue injury. This inflammatory cascade can precipitate acute-on-chronic liver failure (ACLF), a critical syndrome defined by rapid hepatic decompensation and progressive extrahepatic organ failure. ACLF accounts for the majority of liver-related mortality in patients with chronic hepatitis, cirrhosis, and hepatocellular carcinoma (1, 2). Despite its potentially reversible nature, ACLF carries high short-term mortality due to the frequent development of multiorgan dysfunction (3, 4). The initial 7-days period post-onset (termed the “golden window”) (5) represents a pivotal therapeutic opportunity for early diagnosis and intervention to modify disease progression. Timely evaluation of inflammatory status is therefore paramount for prognostic improvement in ACLF.

The phase angle (PhA), derived non-invasively through bioelectrical impedance analysis, correlates with early-phase inflammation and oxidative stress. PhA values exhibit an inverse relationship with the severity of these pathological processes (6). As a composite marker reflecting cellular integrity, membrane stability, and hydration status (7), reduced PhA indicates compromised cellular structure and increased apoptosis (7, 8). Existing evidence demonstrates PhA alterations across various chronic conditions (9–11), yet its clinical significance in ACLF remains undetermined. This study was designed to systematically evaluate the association between PhA and ACLF severity, with particular focus on its potential prognostic utility.

## Materials and methods

### Study participants

This retrospective study collected data from ACLF patients who were hospitalized at our institution from December 1, 2020 to March 1, 2023. The study was approved by the Medical Ethics Committee of Zhongshan Hospital affiliated with Xiamen University (approval number: 2023 - 145). Before initiating any research procedures, we obtained written informed consent from all subjects and their guardians. All methods were executed in strict compliance with the relevant guidelines and regulations.

The inclusion criteria were based on the 2019 diagnostic criteria for ACLF established by the Asia-Pacific Association for the Study of the Liver (APASL) (5). These criteria included patients with chronic hepatic diseases, encompassing those with hepatitis without cirrhosis, those with cirrhosis, and those with primary liver cancer. Additionally, the criteria required serum bilirubin levels ≥ 5 mg/dL, an international normalized ratio (INR) ≥ 1.5, prothrombin activity (PTA) ≤ 40%, and complications such as infection, ascites, and hepatic encephalopathy (HE). Patients with pacemakers, amputations, aneurysm clips, or metal implants were excluded due to potential interference with bioelectrical impedance analysis results. The control group comprised 96 patients: 45 in Control Group 1 had chronic liver disease with normal liver function, and 51 in Control Group 2 had abnormal liver function but did not meet the criteria for liver failure.

### Data collection

Clinical and demographic data, including age, sex, body mass index (BMI), etiology, complications, laboratory parameters, and prognostic scores, were collected. Ascites was assessed and graded by ultrasound. Hepatic encephalopathy was diagnosed according to neuropsychiatric symptoms, blood markers, and neurological signs.

### Bioelectrical impedance analysis

Phase angle was measured using the InBody S10 body composition analyzer and calculated using the formula PhA (°) = arctan (*Xc/R*) × (180°/π), where *Xc* is reactance and *R* is resistance at 50 kHz bioelectrical impedance analysis (BIA). Before measurement, participants were required to fast for ≥ 2 h, refrain from vigorous physical activity and intravenous infusion, and completely empty their bladder and bowels. They rested in a supine position for ≥ 10 min before testing.

For the measurement procedure, subjects assumed a supine position with full exposure of the skin on both hands and ankles. The feet were positioned 10–15 cm apart, and the hands were placed palm-up alongside the body with fingers extended and arms abducted at 15°. The skin at electrode contact sites was cleaned with alcohol swabs. Four electrodes were placed on the distal metacarpals (left/right hands) and metatarsals (left/right ankles). During measurement, subjects were instructed to avoid contact with any extraneous metal objects.

### Statistical analysis

Continuous variables following a normal distribution were expressed as mean ± standard deviation, while non-normally distributed continuous variables were reported as medians (interquartile ranges). Continuous variables were compared between two groups using either Student’s *t*-test or the Mann-Whitney U test, as appropriate. For continuous variables involving two or more groups, comparisons were performed using either one-way analysis of variance (ANOVA) or the Kruskal-Wallis test, as appropriate. Categorical data were analyzed using either the χ^2^ test or Fisher’s exact test. Correlations between variables were assessed using Pearson’s or Spearman’s correlation analysis, depending on data distribution. The optimal cutoff value for predicting 90-days mortality was determined through receiver operating characteristic (ROC) curve analysis, with an area under the curve (AUC) > 0.5 considered indicative of diagnostic value. Statistical significance was set at *P* < 0.05. All analyses were performed using SPSS 26.0 (SPSS Inc., Chicago, Illinois, USA) and GraphPad Prism (version 8.0.2).

## Results

### Patient characteristics

The study included 78 patients with ACLF, including 58 males (74.36%). Control group 1 consisted of 45 patients (30 males, 66.67%), while control group 2 comprised 51 patients (40 males, 78.43%). The groups had comparable sex and age distributions. Ascites severity, assessed by ultrasonography, was graded as: Grade 1 (mild) - detectable only by ultrasound with maximal fluid depth < 3 cm; Grade 2 (moderate) – 3–10 cm of intraperitoneal fluid; and Grade 3 (severe) - fluid collections > 10 cm deep. Clinical indicators, complications, prognostic scores, and PhA were compared between groups (Table 1). The ACLF group showed lower PhA values [4.55° (3.70°–5.45°)] compared to control group 1 [5.90° (5.50°–6.30°); *P* < 0.001] and control group 2 [5.30° (4.60°–6.00°); *P* < 0.001].

**TABLE 1:** Physical characteristics and biochemical measurements of Acute-on-chronic liver failure (ACLF) patients and the controls.

Items	Control 1 (*n* = 45)	Control 2 (*n* = 51)	ACLF (*n* = 78)
Age (years)	52.00 (43.00–60.00)	53.00 (45.00–61.00)	53.00 (44.00–61.25)
Male, (%)	30 (66.67)	40 (78.43)	58 (74.36)
Female, (%)	15 (33.33)	11 (21.57)	20 (25.64)
BMI	24.02 (21.73–25.90)	21.89 (20.07–24.34)	22.30 (20.20–26.18)
**Etiological**			
CHB (%)	45 (100)	26 (50.98)	50 (64.10)
AFLD (%)	0 (0)	7 (13.73)	10 (12.82)
NAFLD (%)	0 (0)	1 (1.96)	2 (2.56)
PhA (°)	5.90 (5.50–6.30)	5.30 (4.60–6.00)	4.55 (3.70–5.45)
ALB (g/L)	44.90 (43.10–46.30)	32.70 (29.70–36.20)	29.54 (26.48–32.25)
TBIL (mg/dL)	0.74 (0.47–0.95)	3.11 (1.61–7.46)	9.75 (6.56–12.93)
ALT (U/L)	25.40 (17.75–37.25)	67.30 (28.50–428.80)	107.25 (38.78–345.30)
AST (U/L)	26.60 (21.65–32.65)	89.80 (47.10–209.40)	132.20 (67.23–358.73)
Na (mmol/L)	140.80 (139.25–141.90)	138.50 (135.90–141.60)	137.90 (135.65–141.15)
CREA (mg/dL)	0.80 (0.66–0.93)	0.74 (0.60–0.84)	0.72 (0.60–0.82)
LDL-C (mmol/L)	3.10 (2.67–3.52)	2.66 (2.04–3.17)	2.23 (1.82–2.70)
INR	1.08 (0.91–1.26)	1.22 (1.09–1.34)	1.69 (1.55–1.90)
PTA (%)	90.22 (82.26–96.35)	60.27 (52.38–78.57)	36.67 (30.45–42.10)
**Complication**			
HE (%)	0 (0)	23 (45.10)	40 (51.28)
No ascites/ Grade 1 ascites/ Grade 2 ascites/ Grade 3 ascites	0/0/0/0	27/14/7/3	20/17/29/12
GIB (%)	0 (0)	5 (9.80)	12 (15.38)
Infection (%)	0 (0)	7 (13.73)	42 (53.85)

BMI, body mass index; CHB, chronic hepatitis B; AFLD, alcoholic fatty liver disease; NAFLD, non-alcoholic fatty liver disease; PhA, phase angle; ALB, albumin; TBIL, total bilirubin; ALT, alanine aminotransferase; AST, aspartate aminotransferase; Na, sodium; CREA, creatinine; LDL-C, low-density lipoprotein cholesterol; INR, international normalized ratio; PTA, prothrombin activity; HE, hepatic encephalopathy; GIB, gastrointestinal bleeding.

### Correlation analysis between ACLF severity and PhA

**FIGURE 1:**
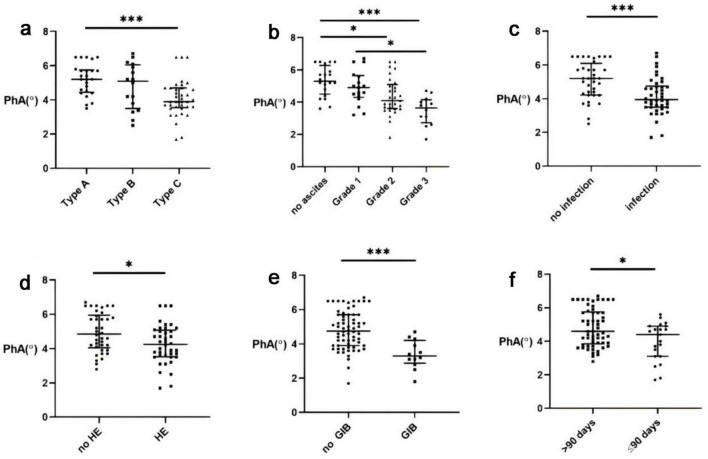
Correlation between Phase angle (PhA) and Acute-on-chronic liver failure (ACLF). **(a)** PhA levels in different types of ACLF. **(b)** ACLF PhA levels and ascites. **(c)** ACLF PhA levels and infection. **(d)** ACLF PhA levels and hepatic encephalopathy (HE). **(e)** ACLF PhA levels and gastrointestinal bleeding (GIB). **(f)** ACLF PhA levels and the 90-days survival rate.*, *P* < 0.05; ***, *P* < 0.001.

Initial analysis revealed significant differences in PhA values among ACLF subtypes. Patients with cirrhotic ACLF exhibited lower PhA values than non-cirrhotic cases, while those with decompensated cirrhosis showed reduced PhA compared to compensated cirrhosis (Figure 1a; *P* < 0.001).

Acute-on-chronic liver failure severity demonstrated an inverse relationship with PhA, with advanced stages showing progressively lower values. The presence of complications - including infection, hepatic encephalopathy, or gastrointestinal bleeding generally exhibited reduced PhA levels (Figures 1c–e; *P* < 0.05). Similarly, patients with ascites had markedly lower PhA than those without, with values declining corresponding to ascites severity (Figure 1b; *P* < 0.001).

**FIGURE 2:**
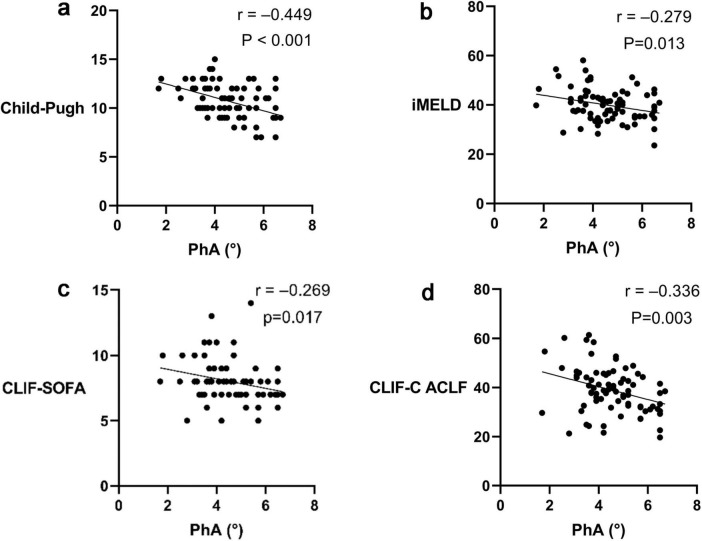
Correlation between Phase angle (PhA) and prognostic scores of Acute-on-chronic liver failure (ACLF). **(a)** Correlation between PhA and the Child-Pugh score. **(b)** Correlation between PhA and the Improved Model for End-Stage Liver Disease (iMELD) score. **(c)** Correlation between PhA and the Chronic Liver Failure - Sequential Organ Failure Assessment (CLIF-SOFA) score. **(d)** Correlation between PhA and the Chronic Liver Failure Consortium Acute - on - Chronic Liver Failure Score (CLIF-C ACLF) score.

Correlation analysis revealed negative associations between PhA and the prognostic scores: Child-Pugh (*r* = −0.449, *P* < 0.001; Figure 2a), Improved Model for End-Stage Liver Disease (iMELD) (*r* = −0.279, *P* = 0.013; Figure 2b), Chronic Liver Failure - Sequential Organ Failure Assessment (CLIF-SOFA) (*r* = −0.269, *P* = 0.017; Figure 2c), and Chronic Liver Failure Consortium Acute - on - Chronic Liver Failure Score (CLIF-C ACLF) scores (*r* = −0.336, *P* = 0.003; Figure 2d).

### Comparison between ACLF survivors and non-survivors

Patients with ACLF were stratified by 90-days survival into survivors (*n* = 57) and non-survivors (*n* = 21). No significant differences were observed in sex ratio, mean age, BMI, or the number of hepatocellular carcinoma cases between the two groups (Table 2).

**TABLE 2:** Analysis of clinical and demographic characteristics between survivors and non-survivors in Acute-on-chronic liver failure (ACLF) (*n* = 78).

Items	Total (*n* = 78)	Survival group (*n* = 57)	Non-survival group (*n* = 21)	*P*-value
Age (years)	52.19 ± 13.71	51.11 ± 13.90	55.14 ± 13.04	0.269
Male (%)	58 (74.36)	41 (71.93)	17 (80.95)	0.630
BMI (kg/m^2^)	22.30 ± 3.87	23.67 ± 3.83	21.90 ± 3.76	0.062
HCC (%)	10 (12.82)	5 (8.77)	5 (23.81)	0.168
Type A/B/C	25/16/37	22/14/21	3/2/16	0.011
**Laboratory data**				
PhA (°)	4.55 (3.70–5.45)	4.60 (3.85–5.75)	4.40 (3.10–4.90)	0.026
ALB (g/L)	29.70 ± 4.84	29.97 ± 4.82	28.95 ± 4.93	0.413
TBIL (mg/dL)	9.75 (6.56–12.93)	9.36 (6.42–12.71)	12.52 (7.05–15.84)	0.086
ALT (U/L)	107.25 (38.78–345.30)	130.40 (40.15–378.40)	57.00 (15.18–208.35)	0.095
AST (U/L)	132.20 (67.23–358.73)	141.70 (63.95–376.85)	108.00 (75.35–233.85)	0.414
Na (mmol/L)	137.68 ± 3.67	138.19 ± 3.72	136.32 ± 3.23	0.046
CREA (mg/dL)	0.72 (0.60–0.82)	0.71 (0.62–0.82)	0.75 (0.55–0.89)	0.919
LDL-C (mmol/L)	2.23 (1.82–2.70)	2.24 (1.80–2.62)	2.16 (1.19–2.84)	0.823
INR	1.69 (1.55–1.90)	1.66 (1.52–2.12)	1.77 (1.64–2.18)	0.026
PTA (%)	36.23 ± 7.75	37.57 ± 7.14	32.60 ± 8.32	0.011
CRP	12.53 (8.43–20.19)	11.82 (8.38–17.64)	14.17 (8.60–46.60)	0.165
**Complications**				
HE (%)	40 (51.28)	25 (43.86)	15 (71.43)	0.022
Ascites none/grade 1/ grade 2/grade 3	20/17/29/12	19/15/20/3	1/2/9/9	0.001
GIB (%)	12 (15.38)	6 (10.53)	6 (28.57)	0.108
Infection (%)	42 (53.85)	25 (43.86)	17 (80.95)	0.004
**Scoring systems**				
Child-Pugh	10.69 ± 1.82	10.25 ± 1.79	11.90 ± 1.30	<0.001
MELD	20.65 ± 3.57	19.97 ± 3.22	22.51 ± 3.90	0.005
MELD-Na	21.45 ± 4.65	20.62 ± 4.16	23.68 ± 5.25	0.009
iMELD	39.94 ± 6.52	38.58 ± 6.60	43.63 ± 4.88	0.002
CLIF-SOFA	7.95 ± 1.52	7.49 ± 1.17	9.33 ± 1.91	<0.001
CLIF-C ACLF	38.83 ± 9.29	36.47 ± 8.28	45.25 ± 8.99	<0.001

Data are expressed as median (IQR), mean ± SD, or number of patients (%). HCC, hepatocellular carcinoma; Type A, acute-on-chronic liver failure emerging from chronic hepatitis without cirrhosis; Type B, acute-on-chronic liver failure occurring on the basis of compensated cirrhosis; Type C, acute-on-chronic liver failure developing from decompensated cirrhosis; PhA, phase angle; HE, hepatic encephalopathy; ALB, albumin; TBIL, total bilirubin; ALT, alanine aminotransferase; AST, aspartate aminotransferase; Na, sodium; CREA, creatinine; LDL-C, low-density lipoprotein cholesterol; INR, international normalized ratio; PTA, prothrombin activity; CRP, C-reactive protein; HE, hepatic encephalopathy; GIB, gastrointestinal bleeding; MELD, Model for End-Stage Liver Disease; MELD-Na, Model for End-Stage Liver Disease-Sodium; iMELD, Improved Model for End-Stage Liver Disease; CLIF-SOFA, Chronic Liver Failure - Sequential Organ Failure Assessment; CLIF-C ACLF, Chronic Liver Failure Consortium Acute - on - Chronic Liver Failure; *P*-values represent comparisons between Survival group and Non-survival group.

The median PhA in the overall ACLF cohort was 4.55° (3.70°–5.45°). Notably, non-survivors demonstrated lower PhA values [4.40° (3.10°–4.90°)] compared to survivors [4.60° (3.85°–5.75°); *P* = 0.026] (Table 2).

The incidence of hepatic encephalopathy, ascites, and infection was significantly higher in non-survivors (all *P* < 0.05). Laboratory analyses revealed non-survivors had significantly higher INR values, lower serum sodium levels, and lower prothrombin time activity compared to survivors (all *P* < 0.05).

Furthermore, non-survivors exhibited significantly elevated scores across all prognostic models, including Child-Pugh, Model for End-Stage Liver Disease (MELD), Model for End-Stage Liver Disease with Sodium (MELD-Na), iMELD, and the CLIF-SOFA (all *P* < 0.05) (Table 2).

### New prognostic model development

Receiver operating characteristic curve analysis demonstrated that phase angle (PhA) alone had moderate predictive value for 90-days mortality in ACLF patients (AUC = 0.665), with a cutoff value of 5.15°, specificity of 90.48%, but limited sensitivity (42.11%) (Table 3). To enhance predictive performance, we developed a composite score combining PhA with the CLIF-SOFA score (Table 4). The novel CLIF-SOFA-PhA model showed improved predictive accuracy (AUC = 0.815; 95% CI: 0.699–0.930) with optimal cutoff at 8.50 (sensitivity 71.43%, specificity 80.70%) (Table 3).

**TABLE 3:** Receiver operating characteristic (ROC) curve for prediction of 90-days mortality.

Models	AUC	Cut off value	95% CI	Sensitivity (%)	Specificity (%)	*P*-value
CLIF-SOFA-PhA	0.815	8.50	0.699–0.930	71.43	80.70	<0.001
PhA	0.665	5.15	0.533–0.796	42.11	90.48	0.026
iMELD	0.758	41.02	0.653–0.863	76.19	71.93	0.001
MELD-Na	0.703	21.30	0.566–0.839	66.67	75.44	0.007
Child-Pugh	0.762	10.50	0.658–0.867	85.71	38.60	<0.001
MELD	0.719	21.30	0.585–0.853	66.67	78.95	0.003
CLIF-C ACLF	0.761	37.88	0.638–0.884	85.71	57.89	<0.001
CLIF-SOFA	0.795	8.50	0.682–0.909	57.14	85.97	<0.001

ACLF, acute-on-chronic liver failure; AUC, area under the curve; CI, confidence interval; ROC, receiver operating characteristic; PhA, phase angle; iMELD, Improved Model for End-Stage Liver Disease; MELD-Na, Model for End-Stage Liver Disease with Sodium; MELD, Model for End-Stage Liver Disease; CLIF-C ACLF, Chronic Liver Failure Consortium Acute - on - Chronic Liver Failure Score; CLIF-SOFA, Chronic Liver Failure - Sequential Organ Failure Assessment.

**TABLE 4:** A new prognostic score for Acute-on-chronic liver failure (ACLF): CLIF-SOFA-PhA.

Organ/system	Testing items	0	1	2	3	4
Liver	Bilirubin, mg/dL	<1.2	≥ 1.2 to < 2.0	≥ 2 to < 6	≥ 6 to < 12	≥12.0
Kidney	Creatinine, mg/dL	<1.2	≥ 1.2 to ≤ 2.0	≥2.0	≥3.5	≥5.0
Cerebral	HE grade	–	I	II	III	IV
Coagulation	INR	<1.1	≥ 1.1 to < 1.25	≥ 1.25 to < 1.5	≥ 1.5 to < 2.5	≥ 2.5 or platelet count ≤ 20 × 10^9^/L
Circulation	MAP, mmHg	≥70	<70	Dopamine ≤ 5 ug/kg/min	Dopamine > 5 ug/kg/min	Dopamine > 15 ug/kg/min
Lung	PaO_2/_FiO_2_ or SpO_2/_FiO_2_	>400 >512	> 300 to ≤ 400 > 357 to ≤ 512	> 200 to ≤ 300 > 214 to ≤ 357	> 100 to ≤ 200 > 89 to ≤ 214	≤100 ≤89
PhA (°)		≥3.5		<3.5		

MAP, mean arterial pressure; PaO_2_, partial pressure of oxygen; FiO_2_, fraction of inspired oxygen; SpO_2_, oxygen saturation; HE, hepatic encephalopathy; PhA, phase angle; INR, international normalized ratio.

Comparative analysis showed that the new CLIF-SOFA-PhA model achieved higher predictive accuracy than existing scoring systems, including MELD-Na (AUC = 0.703; 95% CI: 0.566–0.839), MELD (AUC = 0.719; 95% CI: 0.585–0.853), iMELD (AUC = 0.758; 95% CI: 0.653–0.863), Child-Pugh (AUC = 0.762; 95% CI: 0.658–0.867), CLIF-C ACLF (AUC = 0.761; 95% CI: 0.638–0.884) and CLIF-SOFA (AUC = 0.795; 95% CI: 0.682–0.909) for predicting 90-days mortality in ACLF patients (Table 3).

## Discussion

The liver, as the body’s primary anabolic organ, plays a central role in maintaining nutritional homeostasis and modulating inflammatory responses. Accumulating evidence supports the clinical utility of PhA as a prognostic indicator in hepatic disorders. In overweight populations, PhA shows consistent association with non-alcoholic fatty liver disease (NAFLD) risk, with NAFLD patients demonstrating reduced PhA values (12). For chronic hepatitis C infection, PhA serves as a predictor of fibrosis progression (13). In cirrhotic patients, PhA values ≤ 4.9°correlate with increased hepatic encephalopathy risk (14), while values ≤ 5.52° show predictive value for 6-months mortality in decompensated cirrhosis (15). While these established associations highlight PhA’s clinical relevance across multiple liver diseases, its potential role in ACLF remains poorly characterized, representing an important area for further investigation.

Our study demonstrates that PhA, a readily measurable biomarker reflecting inflammatory status, serves as a valuable indicator for assessing disease severity and predicting 90-days survival in ACLF.

Initial measurements revealed PhA values of 5.90° (5.50°–6.30°) in patients with chronic liver disease and normal liver function, consistent with previous reports (16). However, these prior studies did not investigate PhA changes during progression from chronic liver disease to ACLF, nor its prognostic value in ACLF.

Our findings provide novel insights into this relationship. We observed a progressive decline in PhA values corresponding to worsening liver function: decreasing to 5.30° (4.60°–6.00°) in chronic liver disease with abnormal function, and further declining to 4.55° (3.70°–5.45°) upon progression to liver failure. These changes likely reflect alterations in cellular integrity and tissue composition associated with disease progression.

Subgroup analysis showed that ACLF patients with cirrhosis had lower PhA values than non-cirrhotic cases, with decompensated cirrhosis patients exhibiting more pronounced reductions compared to compensated cases (Figure 1). These differences may relate not only to liver disease severity but also to the frequent occurrence of sarcopenia in cirrhosis (17). A 2021 study by Ruiz-Margáin A et al. reported that PhA effectively identifies sarcopenia in cirrhosis with diagnostic accuracy comparable to skeletal muscle index (SMI) (18).

Additional analyses demonstrated an inverse relationship between PhA and ACLF severity. Patients with complications exhibited significantly lower PhA values than those without (Figure 1). This observation aligns with PhA’s potential role as an inflammatory biomarker, reflecting both systemic inflammatory activity and cellular dysfunction. The physiological basis involves characteristic bioelectrical changes during inflammation: reduced cell membrane capacitance decreases capacitive reactance (Xc), while expanded extracellular water content lowers resistance (R). Since PhA represents the Xc/R ratio, and given Xc’s greater relative decline, the net effect is reduced PhA values that correlate with inflammatory burden and cellular damage extent (6).

Consequently, PhA is regarded as a predictive indicator of disease severity (19), and it is believed that the strength of the inflammatory response determines ACLF severity (20).

Comparative analysis of ACLF survivors and non-survivors indicated an association between reduced PhA values and more advanced disease severity, with lower measurements tending to correlate with poorer outcomes. These findings are consistent with previous reports establishing PhA as a potential prognostic marker for short-term mortality. For instance, Cornejo-Pareja et al. compared the PhA values in 127 COVID-19 patients between survivors and non-survivors and found a significant difference (survivors: 4.5° (3.5°–5.5°), non-survivors: 2.8° (2.08°–3.68°), *P* < 0.001) (21). Similarly, Bernal-Ceballos et al. reported that acute heart failure patients with admission PhA values below 4.8° experienced higher 90-days mortality rates (22).

Several clinical scoring systems are currently available for assessing ACLF severity and prognosis, including the Child-Pugh, MELD, MELD-Na, iMELD, and CLIF-SOFA scores, though each has notable limitations (23). Previous research by Pagano et al. identified PhA as a potential prognostic marker in chronic liver diseases, particularly cirrhosis and hepatocellular carcinoma, with values below 5.1° associated with reduced 15-months survival in liver cancer patients (24). Additionally, lower PhA is associated with malnutrition and prolonged hospitalizations in critically ill patients in the intensive care unit (25).

In contrast to commonly used severity scoring systems in the intensive care unit, such as the Acute Physiology and Chronic Health Evaluation (APACHE), Sequential Organ Failure Assessment (SOFA), and Simplified Acute Physiology Score (SAPS), PhA demonstrates a stronger predictive ability for mortality among critically ill patients (19). The prognostic utility of PhA in chronic liver disease and critical illness may derive from its reflection of underlying inflammatory and oxidative stress pathways (6, 26).

Building upon existing evidence, we examined whether PhA might predict 90-days survival in ACLF patients. Our analysis showed that PhA alone yielded an AUC of 0.665 (95% CI: 0.533–0.796) for predicting 90-days mortality, with 42.11% sensitivity and 90.48% specificity. While PhA demonstrated limited sensitivity for mortality prediction, its high specificity may be clinically useful. For patients with ambiguous biochemical markers, maintained PhA values could suggest more favorable short-term outcomes.

The CLIF-SOFA scoring system evaluates the functional status of major organs and aggregates scores to predict mortality within 28 and 90 days in ACLF patients. Developed primarily for Western populations with alcohol- or HCV-related cirrhosis, its application may be less optimal for HBV-predominant Asian-Pacific cases. Hence, the CLIF-SOFA scoring system requires further optimization for the Asia-Pacific region. In our data, CLIF-SOFA also demonstrated high prognostic value (AUC = 0.795); however, the system lacks inflammation-related parameters relevant to the early phases of the disease. In ACLF, inflammatory responses play a crucial role, and the onset of an inflammatory storm occurs before organ function impairment. Incorporating easily detectable and accurate inflammation-related indicators into the CLIF-SOFA scoring system could enhance the early prediction of ACLF prognosis.

Our study observed differences in PhA values between ACLF survivors and non-survivors. In comparison, C-reactive protein (CRP) levels showed less pronounced variation between groups. This may be attributed to CRP’s hepatic origin - in liver failure, compromised synthetic function appears to reduce CRP production. As documented in previous studies (27, 28), even during sepsis-associated fulminant liver failure with marked inflammatory stimulation, CRP levels tend to remain low. These findings indicate PhA may offer advantages over CRP in monitoring inflammatory responses in ACLF.

To improve prognostic accuracy, we developed a novel CLIF-SOFA-PhA composite score. ROC analysis demonstrated improved performance (AUC = 0.815, sensitivity 71.43%, specificity 80.70%) compared to existing scores [MELD (AUC = 0.719), MELD-Na (AUC = 0.703), iMELD (AUC = 0.758), Child-Pugh (AUC = 0.762), CLIF-C ACLF (AUC = 0.761), and CLIF-SOFA (AUC = 0.795)], indicating that combining organ failure and inflammatory immune response indicators may improve prediction of 90-days mortality in ACLF.

Our research had certain limitations. First, it was a single-center retrospective analysis with a limited sample size, therefore, validation work needs to be conducted in a larger patient population in the future. Second, the study population was based on Chinese individuals, with most cases involving chronic hepatitis B or hepatitis B-associated cirrhosis. Whether these findings apply to other ethnic backgrounds and etiologies of ACLF remains to be validated. Moreover, PhA may undergo dynamic changes during the rapid evolution of ACLF.

Therefore, future research should thoroughly address these limitations by collecting larger-scale data, including different ethnic groups, and observing the variations of PhA during the progression of ACLF. This will further enhance our understanding of the disease and assist in refining clinical treatment decisions.

## Data Availability

The original contributions presented in this study are included in this article/supplementary material, further inquiries can be directed to the corresponding author.
