# Correction: Phase angle associates with severity and mortality in acute-on-chronic liver failure

**DOI:** 10.3389/fmed.2025.1695643

**Published:** 2025-10-14

**Authors:** Shiran Cai, Liqun Lin, Yanyan Cai, Chenhao Wang, Yufen Lin, Jingping Zhou, Fei Zhou, Meiya Chen

**Affiliations:** ^1^Department of Gastroenterology, The National Key Clinical Specialty, Zhongshan Hospital of Xiamen University, School of Medicine, Xiamen University, Xiamen, Fujian, China; ^2^The Graduate School of Fujian Medical University, Fuzhou, China; ^3^Department of Gastroenterology, The Second Affiliated Hospital of Fujian Medical Uiversity, Quanzhou, Fujian, China; ^4^Department of Clinical Nutrition, Zhongshan Hospital of Xiamen University, School of Medicine, Xiamen University, Xiamen, Fujian, China; ^5^Department of Oncology, Cancer Hospital, Fudan University (Xiamen Branch), Xiamen, Fujian, China; ^6^Institute for Microbial Ecology, School of Medicine, Xiamen University, Xiamen, Fujian, China; ^7^Department of Digestive Disease, School of Medicine, Xiamen University, Xiamen, Fujian, China; ^8^Xiamen Key Laboratory of Intestinal Microbiome and Human Health, Zhongshan Hospital of Xiamen University, School of Medicine, Xiamen University, Xiamen, Fujian, China

**Keywords:** acute-on-chronic liver failure, phase angle, prognosis, inflammation, bioelectrical impedance analysis

Affiliation “Department of Gastroenterology, The National Key Clinical Specialty, Zhongshan Hospital of Xiamen University, School of Medicine, Xiamen University, Xiamen, Fujian, China” was erroneously given as the fifth affiliation.

The original version of this article has been updated.

